# COVID-19 epidemic in remote areas of the French Amazon, March 2020 to May 2021: Another reality

**DOI:** 10.1590/0037-8682-0274-2021

**Published:** 2022-04-29

**Authors:** Loïc Epelboin, Tiphanie Succo, Céline Michaud, Margot Oberlis, Bastien Bidaud, Pauline Naudion, Lise Dudognon, Clara Fernandes, Charlène Cochet, Cécile Caspar, Estelle Jacoud, Sébastien Teissier, Maylis Douine, Dominique Rousset, Claude Flamand, Félix Djossou, Mathieu Nacher, Cyril Rousseau, Nicolas Vignier, Mélanie Gaillet

**Affiliations:** 1 Centre Hospitalier de Cayenne Andrée Rosemon, Unité des Maladies Infectieuses et Tropicales, Cayenne, French Guiana.; 2 Centre Hospitalier de Cayenne Andrée Rosemon, Centres Délocalisés de Prévention et de Soins, Cayenne, French Guiana.; 3 Centre Hospitalier de Cayenne, Centre d’investigation clinique Antilles Guyane, CIC Inserm 1424, DRISP, Cayenne, French Guiana.; 4 Santé Publique France, Cayenne, French Guiana.; 5 Croix Rouge Française, Cayenne, French Guiana.; 6 Centre Hospitalier de Cayenne, Centre de Santé de Saint Georges de l’Oyapock, Cayenne, French Guiana.; 7 Centre Hospitalier de l’Ouest Guyanais, Service de médecine et maladies infectieuses, Saint Laurent du Maroni, French Guiana.; 8 Centre Hospitalier de Cayenne Andrée Rosemon, Equipe Mobile de Santé Publique en Commune, 97300 Cayenne, French Guiana.; 9 Centre Hospitalier de Cayenne, Centre de Santé de Maripasoula, French Guiana .; 10 Institut Pasteur de la Guyane, Centre National des Arbovirus, Cayenne, French Guiana.; 11 Institut Pasteur de la Guyane, Unité d’Epidémiologie, Cayenne, French Guiana.; 12 Sorbonne Université, Institut Pierre Louis d’Épidémiologie et de Santé Publique, Department of social epidemiology, IPLESP, Inserm UMR 1136, Paris, France.

**Keywords:** French Guiana, COVID-19, SARS-CoV-2, COVID-19 vaccines, Health promotion

## Abstract

**Background:**

French Guiana (FG) is an ultra-peripheral European region in the Amazon, and the COVID-19 epidemic has had very different kinetics from both its giant neighbors, Brazil or mainland France.

**Methods:**

This study summarized the epidemics of COVID-19 in FG.

**Results:**

The tropical climate, multiethnicity, and remoteness of the population forced healthcare providers to accordingly adapt the management of the epidemic. Incidence and mortality have been lower than that in Europe and Latin America due to a combination of prevalence of the youth in the population and highly developed healthcare system.

**Conclusions:**

Currently, vaccine hesitancy hinders the rapid expansion of vaccine coverage.

French Guiana (FG) is an ultra-peripheral European region in the Amazon on the northeastern shore of South America, nestled between Suriname in the west and the Brazilian state of Amapá in the east. The legal population was estimated at 290,691 inhabitants in January 2021 (https://www.insee.fr/fr/accueil), but this figure does not reflect a reality where many people illegally reside within the territory (in the forest, gold diggers, mostly from Brazil, and, on the coast, slum dwellers around the main cities, mainly from Haiti). FG is characterized by great ethnic and cultural diversities, with varied specificities from one village to another. The cities along the coast are melting pots of Creole Guianese, West Indian, mainland French, Brazilian, Chinese, Surinamese, Dominicans, Haitians, Peruvians, Guyanese, and Hmong, whereas villages in the interior have pronounced ethnic identities (Amerindians, Maroons, and Brazilians). The Amerindians of FG are represented by six ethnic groups, distributed mainly along the two Guianese border rivers: the Maroni in the west and the Oyapock in the east. They share the shore of the Maroni and Lawa rivers with the Maroons, descendants of runaway slaves from neighboring Suriname. The public health system is based on three hospitals on the coastal strip, in Cayenne, Kourou, and Saint Laurent du Maroni, and 17 Remote Centers of Prevention and Care (RCPC) coordinated by the Cayenne hospital and organized around three pivotal centers of Saint Georges de l'Oyapock in the east and Maripasoula and Grand Santi in the west (Figure 1).

**FIGURE 1:**
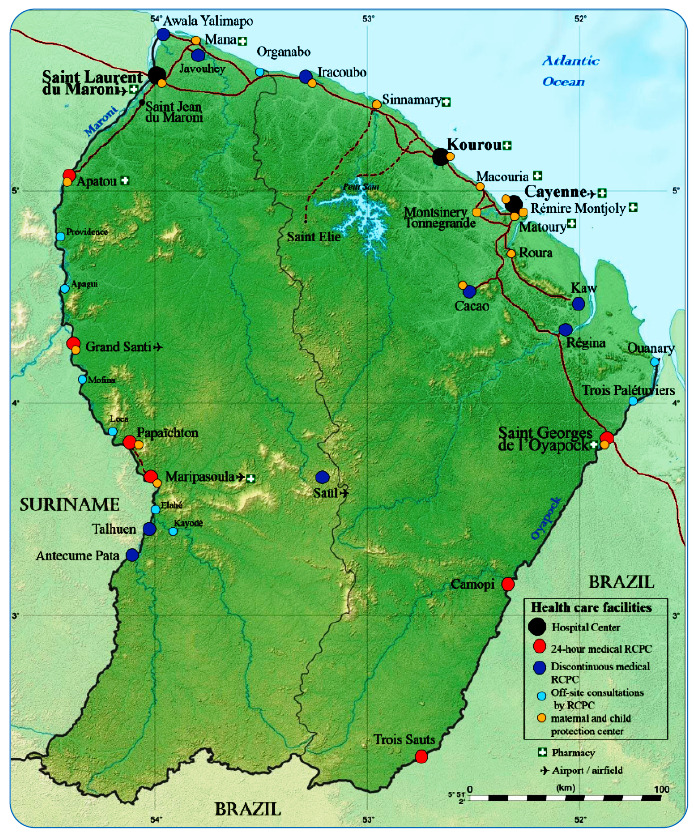
Map of French Guiana with details of the healthcare facilities. RCPC, Remote Centers of Prevention and Care.

TheSARS-CoV-2 epidemic in FG was different from that in Europe and mainland France. This study aimed to describe the specificities of the COVID-19 epidemic between March 2020 and April 2021 in FG, particularly among indigenous people in isolated municipalities, and to describe the implementation of vaccination.

The FG population was locked down at the same time as mainland France on March 17, 2020, although there were only a handful of COVID-19 cases (mainly imported from France and the West Indies)^1^. These cases represented the first epidemic “wavelet” in FG with approximately 120 cases in 2 months^2^. However, in mid-May, while the European wave was ebbing, the first real wave of COVID-19, with high community transmission, reached FG from the east, through the porous border with Brazil. The epidemic severely affected the neighboring state of Amapá^3^ and reached most of the villages of FG successively, and sometimes concomitantly (https://www.portal.ap.gov.br). Thousands of cases occurred within a few weeks, with a maximum of 1,400 cases (471 cases/100,000 inhabitants) in 2 weeks in early July and approximately 9,200 cumulated cases at the beginning of September 2020, heterogeneously distributed throughout the territory (Table 1). In response to this health crisis, piecemeal measures have been implemented, as along with the provision of equipment and human resources. These measures were coordinated by the regional health authorities and Cayenne hospital, which manages the RCPC. They have been adapted from one village to another following epidemic trend. The measures for dealing with the epidemic increasingly diverged from practices in mainland France and EU to adapt to the specificities of FG: localized confinement with curfews of increasing severity, limitation of domestic flights from mainland France and local flights from March 2020(main link for the municipalities of the interior) and limitation of population movements across the territory, strict border closures and control on arrival of air and land travelers with “checkpoints” between the main Guianese cities along the coast. The combination of these measures coincided with a reduction in the basic reproduction number of SARS-CoV-2by 36%, from 1.7 prior to interventions to 1.1 following their implementation, which was sufficient to avoid hospital saturation^1^. Some municipalities paid a heavy social price: prolonged restriction of freedom, worsening of the precariousness of populations already in great difficulty, and mental health impact. Some villages reported a high number of hospitalized patients (e.g., Grand Santi). Others reported a few deaths (e.g., Maripasoula). However, the situation in FG was strikingly different from the great difficulties encountered in the Brazilian neighbors in terms of the number of cases, as in Amapá with 120,403 cumulative cases by the end of July 2021 (1,892 deaths), or in terms of lethality, as described in the Amazonas, during the same period (414,000 cumulative cases and 13,482 deaths)^4^ ^,^ ^5^ (Table 2). In FG, during the first wave, approximately 1,700 patients (584 cumulative hospitalizations/100,000 inhabitants) were hospitalized, including 137 patients (47/100,000 inhabitants) in intensive care and 59 deaths (20/100,000 inhabitants), with an in-hospital lethality of 3.5%.

**TABLE 1:** Comparison of cumulative numbers and incidence rates of cases and deaths March 1, 2020,to July 31, 2021, of French Guiana and closer Brazilian states Amapá and Amazonas.

	Population	Cumulative number of cases	Cumulative number of deaths	Incidence rate per 100,000 population	Mortality per 100,000 population	Case fatality rate per 1,000 cases
French Guiana*	294,071*	9,200	186	3,165	20	6
Amapá**	845,731	120,403	1,892	14,237	224	16
Amazonas**	3,807,923	414,000	13,482	10,872	354	33

*January, 2021 French census; **2019 Brazilian census.

**TABLE 2:** Comparison of cumulative numbers of positive tests, positivity rate, cumulated incidence and mortality rates in various regions of French Guiana, March 1, 2020, to July 31, 2021.

	Towns	Cumulated number of positive tests	Positivity rate	Cumulated incidence rate (per 100,000 inhabitants)	Cumulated mortality rate (per 100,000 inhabitants)
Cayenne and surroundings	Cayenne, Rémire-Montjoly, Matoury, Montsinery-Tonnégrande	15,922	9%	12,586	81
“Savanes”	Kourou, Tonate-Macouria, Sinnamary, Iracoubo	5,626	9%	12,306	68
West	Mana (including Javouhey), Awala-Yalimapo, Saint Laurent du Maroni	5,025	11%	8,555	61
Maroni	Apatou, Grand Santi, Papaïchton, Maripasoula	1,563	19%	4,289	19
Oyapock	Ouanary, Saint Georges de l’Oyapock, Camopi (including Trois Sauts)	1,075	21%	16,916	31
Other	Regina (including Kaw), Roura (including Cacao), Saint Elie, Saül	268	6%	5,686	0
French Guiana		29,479	10%	10,141	65

The inhabitants of Saint Georges de l'Oyapock, a village on the Brazilian border, remained in trouble for several months, as the town has been subjected to strict travel restrictions without interruption since March 17, 2020-nearly a year-including a travel ban to the main town Cayenne. The lives of the populations with mixed ethnicities, Palikur Amerindians, Creoles, Europeans, and Brazilians, who live on both sides of the border river, were particularly affected. In this village with approximately 5,000 inhabitants, around 450 cases were confirmed during the first wave. Some villages were less affected, either in terms of number of cases, as in the Teko and Wayãpi Amerindian populations of the Upper Oyapock (around 70 cases confirmed among 1,800 inhabitants of Camopi), or in terms of clinical severity, as in the Wayana (Amerindians) or Maroon populations (Aluku and Ndjuka) on the Upper Maroni (around 700 cases confirmed among 37,000 inhabitants from Apatou to Maripasoula). The low impact in these communities was reassuring because, at the same time, disturbing data were being published on the heavy burden that Brazil’s Amerindian communities were paying^6^. A peak associated with a regional seroprevalence of 15.4% was reached in early July, and the situation calmed down, allowing the gradual relaxation of the restrictive measures in place from the end of July to the end of September 2020 (Figure 2) (https://www.santepubliquefrance.fr/). As the second wave arrived in the UE and mainland France at the end of October, leading to a new curfew, FG seemed to have minimal viral circulation.

**FIGURE 2:**
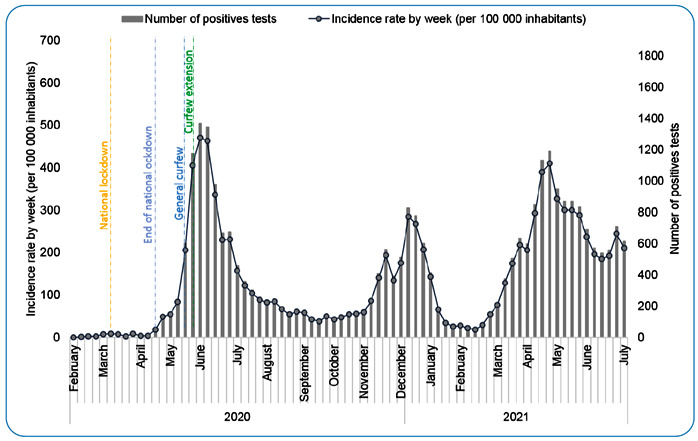
Incidence rate (per 100,000 inhabitants) and weekly number of cases of COVID-19 in French Guiana, March, 2020 to April, 2021.

The epidemic spread widely among the populations of the interior territories with an estimated seroprevalence ranging from 1% to 35% in September 2020, between Cacao and Maripasoula, respectively, at the end of the first real wave^7^. The number of severe cases and deaths has been relatively low (lethality of 34 deaths/100,000 inhabitants in FG [98 deaths on April 22, 2021] vs. 152 deaths/100,000 inhabitants in mainland France [101,597 deaths on April 22, 2021], with a total of <10 deaths in Maroni and Oyapock). For a population of approximately 6,161 inhabitants in Oyapock (https://www.insee.fr/fr/accueil), the geographical remoteness of the populations generated significant tensions, particularly in the transfer of COVID-19 patients to the coast, only where hospital care is possible, to date (https://www.worldometers.info/coronavirus/). The concerned villages are geographically distant; thus, helicopter transfer is the main alternative for urgent medical evacuations, sometimes replaced by plane or canoe.

At the end of November, 2020, the number of cases and the positivity rate increased once more, in connection with active epidemics in northern Brazil and mainland France, outlining a second wave. This was similar to the epidemic rebound observed in previous months in mainland France and in most countries worldwide. The increase in incidence peaked in early January before decreasing, particularly after the curfew was reinstated and the authorities cancelled the carnival, a major cultural tradition in FG. At the end of February, the stabilization of the epidemic, with an incidence below 50/100,000 inhabitants, allowed a further easing of restrictions with a reduction in curfew hours. This second wave was half as high as that of the first wave: approximately 5,700 cases were confirmed, including 800 per week during the epidemic peak.

At the beginning of March, 2021, while the alpha variant of concern (VOC) was raging in Europe, and the gamma VOC was spreading in Northern Brazil, the number of positive cases for COVID-19 remained low in FG^8^ ^,^ ^9^. Thus, at the end of February, no VOC was detected (10). After 2 weeks, 18 alpha and 27 gamma VOCs were detected. The proportion of variants, especially the gamma VOC, among the positive cases tested through sequencing, evolved very rapidly within a few weeks: January, 0%; February, 16%; March, 73%;and April, 96% of all sequenced cases in FG, leading to a further strengthening of containment measures (https://solidarites-sante.gouv.fr)^10^ ^,^ ^11^ ^,^ ^12^. A third wave, mainly due to the gamma variant, had been rolling in FG until July2021 and was followed without a real decrease by the fourth wave due to the delta VOC, which made the gamma VOC quickly disappear by September, 2021 and has been continuing until now, with a daily incidence rate > 400/100,000 inhabitants (Figure 2). Although mutations acquired by the variant, which affect the spike protein, the target of vaccine immunity, are likely to alter vaccine efficacy, preliminary in vitro data suggest that mRNA vaccines, Pfizer/BioNtech® will remain effective against this variant, albeit with a loss of the antibody neutralizing capacity^13^. French authorities decided not to use the AstraZeneca® vaccine in FG because of its apparent lower efficacy against the gamma variant^14^. Thus, while immunization was only proposed in mainland France to people over 75 years of age and people with comorbidities and caregivers older than 50 years, in February 2021, different decisions were made in FG with the objective of mitigating the impact of the third wave. After an initial application of the French criteria, the indication for vaccination was extended to those over 50 years of age (February 15, 2021) and to all caregivers and to be finally extended to those over 30 years of age (March 29), over 18 years of age (March 19), and over 12 years of age (June).

The global challenge lies in the rapid vaccination of the greatest possible number of people. Bringing vaccines to health facilities is also challenging given the distances and means of transport required. In the FG, the issue is sensitive to remote areas. Since March 2020, the activities and logistics of the RCPC have been strongly impacted by the implementation of screening and secure patient care. Health mediation has been a pillar of these “outbound” activities, with home follow-up of fragile, positive patients, but, above all, on health promotion and the encouragement of screening. The hiring and training of health mediators have played a fundamental role in optimizing the health paths of these populations. Today, the implementation of vaccination also relies on these skills, the absence of which would make the link between the health system and local populations impossible. Indeed, mistrust towards vaccination is unfortunately very strong in the interior: two-dose vaccination coverage of 13% in the Upper Oyapock sector and 7% in the Maroni sector at the end of July^15^. It is driven by social beliefs amplified by fake news and conspiracy theories. Rumors have been circulating about the authorities’ desire to conduct experiments on Maroons or on Amerindians, not to mention other rumors spread globally, notably about the possible risk of transformation of inoculated humans into mutants, the risk of sterility, or the risk of injecting fleas as well as nanoparticles. More reasonably, the Wayana Amerindian and Maroon traditional chiefs considered that they were massively contaminated during the first wave, without paying a heavy price to COVID-19 in terms of serious disease outcomes and deaths, and remain doubtful about the seriousness of this imported disease. They also consider that the traditional preventive pharmacopoeia protects them from severe infections, as observed in other settings and for other diseases^16^. These beliefs cast serious doubts on the interest in vaccination in their communities.

Whereas in mainland France and the EU, people who want to be vaccinated anxiously wait for elusive appointment dates, in FG, there is a lukewarm enthusiasm for vaccination in the general population, both on the coast and in isolated villages along the two border rivers. Furthermore, at the end of September, 2021, a real gap appeared within the civil society between pro-vaccination and anti-vaccination, at the origin of numerous incidents and more sustained and aggressive demonstrations, together with obtaining the lowest rate of vaccination coverage of the whole French territory. Nonetheless, trained teams of caregivers and health mediators actively visit the isolated Amerindian and Maroon communities, as well as the poorest and most socially vulnerable populations along the coast. They fight against prejudice and provide clear information adapted to the level of literacy and the culture of the different populations and convince the most skeptical in the interest of a major collective vaccination effort, through information, awareness, and “going back and forth” between populations and the health authorities, rushing as the arrival of the gamma and delta VOC slooms.
